# 2436. Infections In Patients Undergoing Extracorporeal Membrane Oxygenation (ECMO) At A Tertiary Centre In India

**DOI:** 10.1093/ofid/ofad500.2055

**Published:** 2023-11-27

**Authors:** Gattu Santosh, Suneetha Narreddy, Vishnu Rao Polati, Ravikiran Barigala, venkat ramesh, niranjan panigrahi, ratnamani sharma

**Affiliations:** Apollo Hospitals, hyderabad, Telangana, India; Apollo Health City, Hyderabad, Telangana, India; apollo hospitals, hyderabad, Telangana, India; Apollo Health City, Hyderabad, Hyderabad, Telangana, India; apollo hospitals, hyderabad, Telangana, India; apollo hospitals, hyderabad, Telangana, India; apollo hospitals, hyderabad, Telangana, India

## Abstract

**Background:**

Infections are a major cause of mortality among patients receiving ECMO treatment. However, little is known about the prevalence and spectrum of infections affecting ECMO patients in India and the factors that contribute to increased risk of infections and mortality in these cases.

**Methods:**

This study presents a retrospective analysis of ECMO patients admitted between 2018-2022. The study recorded demographic information, ECMO indication, duration, type of infection, and outcomes. The primary objective of the study was to determine the prevalence of microbiologically-proven nosocomial infections. The study also aimed to identify secondary factors associated with an increase in the number of infections and mortality.

**Figure:**
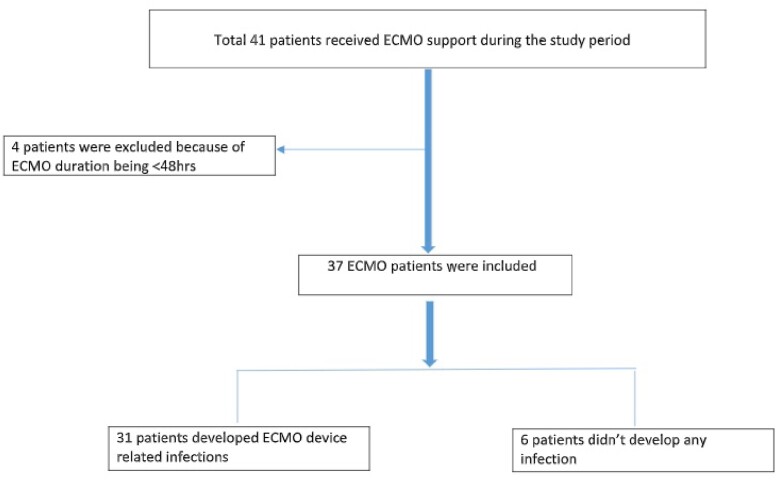

**Results:**

During the study period, 37 patients received ECMO support, with a mean age of 44.97±14.19 years. The most common indication for ECMO was respiratory failure secondary to ARDS. 26 (70.3%) patients were placed on VV-ECMO, while 11 (29.7%) patients received VA-ECMO. 29 patients (83.4%) developed infections, with an incidence of 147.1 infections per 1000 ECMO days. Ventilator-associated pneumonia (48.04%) was the most frequently reported type of infection, followed by bloodstream infection (47.1%). The most commonly isolated organisms were *Klebsiella pneumoniae* (27%), *Acinetobacter baumanii* (15%) and *B. Cepacia* (13%) with *Staphylococcus* and *Enterococci* accounted for only 10% of infections. Additionally, carbapenem resistance was found in 80% and 100% of *Klebsiella* and *Acinetobacter* isolates respectively. Candidemia accounted for 10% of infections, with one patient developing candidemia as early as 4 days after ECMO initiation. Our study revealed a mortality rate of 62% among those who developed infections versus 51% in those who did not develop infections. Patients who developed infections more frequently required CRRT (62% vs. 12.5%, p =0.013). Longer the duration of ECMO, greater the number of infections were observed ( < 7d- 24%; 7-14d- 33.65% & >14d- 42.3%) (Table-4).

Figure-1

**Figure:**
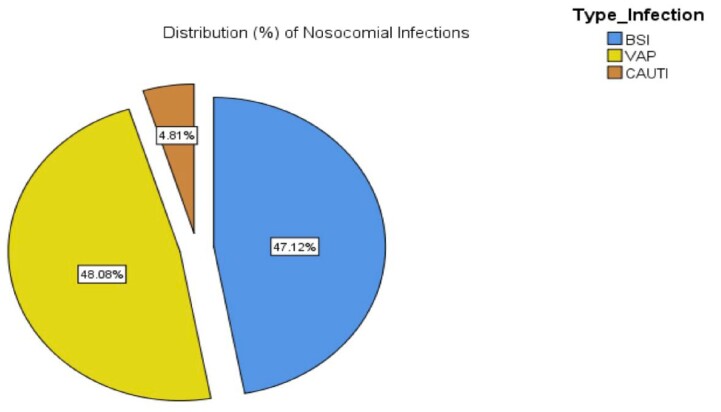

Distribution (%) of Nosocomial infections

Figure-2

**Figure:**
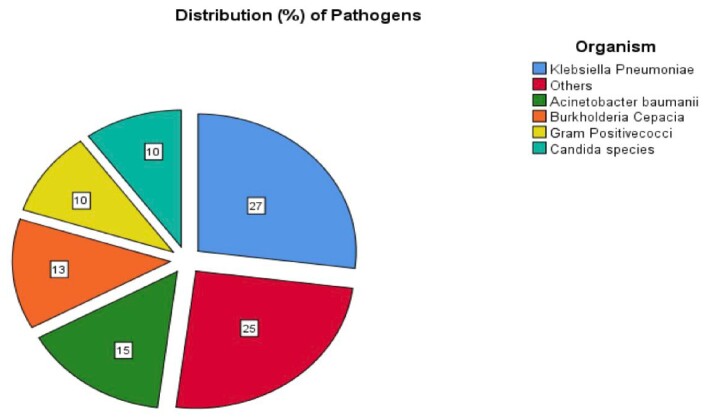

Distribution (%) of Pathogens

Table-2

**Figure:**
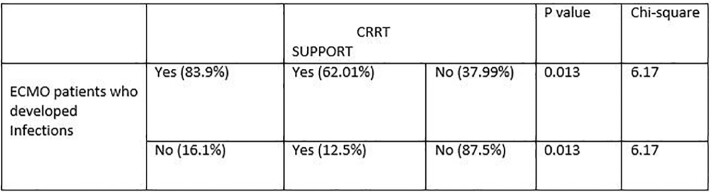

Requirement of CRRT among ECMO patients

**Conclusion:**

There was a high incidence of infections (particularly due to carbapenem resistant organisms) in ECMO patients in our study. Infection risk correlated with duration of ECMO and need for CRRT.

Table-3

**Figure:**
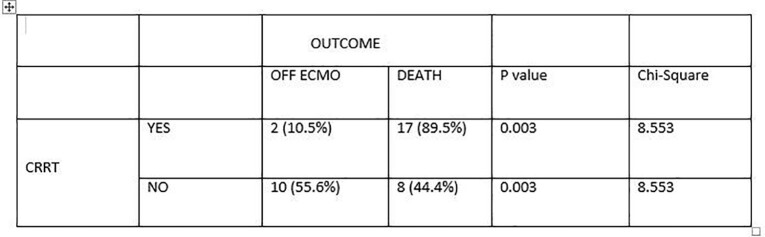

Correlation between CRRT & Mortality

Table-4

**Figure:**
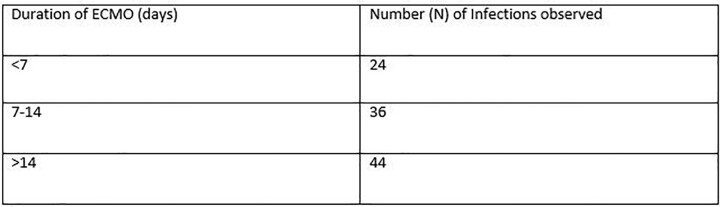

Correlation of number of infections with ECMO Duration

**Disclosures:**

**All Authors**: No reported disclosures

